# Endothelial Progenitor Cells in Neurovascular Disorders—A Comprehensive Overview of the Current State of Knowledge

**DOI:** 10.3390/biomedicines10102616

**Published:** 2022-10-18

**Authors:** Ewa Rudnicka-Drożak, Paulina Drożak, Grzegorz Mizerski, Martyna Drożak

**Affiliations:** 1 Department of Family Medicine, Medical University of Lublin, Langiewicza 6a, 20-035 Lublin, Poland; 2 Student Scientific Society, Department of Family Medicine, Medical University of Lublin, Langiewicza 6a, 20-035 Lublin, Poland

**Keywords:** endothelial progenitor cells, endothelial dysfunction, Alzheimer disease, ischemic stroke, migraine

## Abstract

Endothelial progenitor cells (EPCs) are a population of cells that circulate in the blood looking for areas of endothelial or vascular injury in order to repair them. Endothelial dysfunction is an important component of disorders with neurovascular involvement. Thus, the subject of involvement of EPCs in such conditions has been gaining increasing scientific interest in recent years. Overall, decreased levels of EPCs are associated with worse disease outcome. Moreover, their functionalities appear to decline with severity of disease. These findings inspired the application of EPCs as therapeutic targets and agents. So far, EPCs appear safe and promising based on the results of pre-clinical studies conducted on their use in the treatment of Alzheimer’s disease and ischemic stroke. In the case of the latter, human clinical trials have recently started to be performed in this subject and provided optimistic results thus far. Whereas in the case of migraine, existing findings pave the way for testing EPCs in in vitro studies. This review aims to thoroughly summarize current knowledge on the role EPCs in four disorders with neurovascular involvement, which are Alzheimer’s disease, cerebral small vessel disease, ischemic stroke and migraine, with a particular focus on the potential practical use of these cells as a treatment remedy.

## 1. Introduction

**Figure 1:**
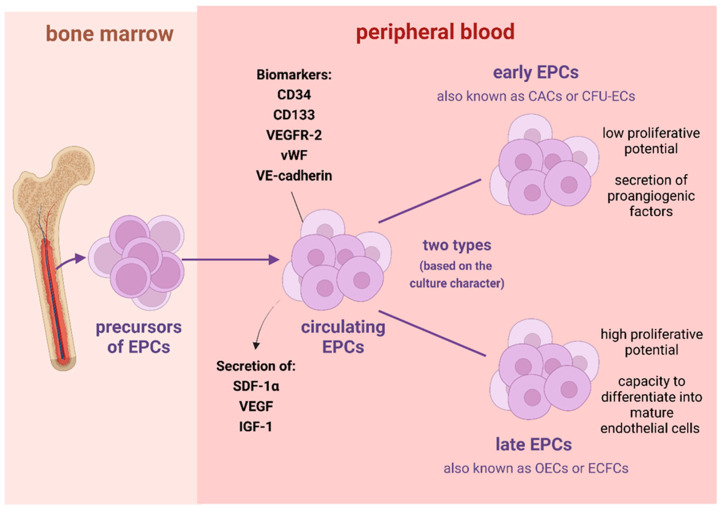
Characteristics of endothelial progenitor cells. Abbreviations: CACs–circulatory angiogenic cells; CFU-ECs–colony-forming unit endothelial cells; ECFCs–endothelial colony-forming cells; EPCs–endothelial progenitor cells; IGF-1–insulin-like growth factor 1; OECs–outgrowth endothelial cells; SDF-1α–stromal cell-derived factor-1α; VE-cadherin–vascular endothelial cadherin; VEGF–vascular endothelial growth factor; VEGFR-2–vascular endothelial growth factor receptor-2; vWF–von Willebrand factor. Image created with biorender.com; accessed on 22 September 2022.

Endothelial progenitor cells (EPCs) are a rare population of cells that originate from the bone marrow [1,2]. They circulate in the peripheral blood looking for areas of endothelial or vascular injury. EPCs have a capacity to repair or replace the damaged endothelium through a differentiation into mature endothelial cells, which are able to embed into the new vessels [3,4]. Moreover, through a secretion of various growth factors, including stromal cell-derived factor-1α (SDF-1α), vascular endothelial growth factor (VEGF) and insulin-like growth factor 1 (IGF-1), they promote angiogenesis or vasculogenesis and recruit more EPCs [5,6]. EPCs express various cell markers on their surface, which include both markers characteristic for hematopoietic stem cells (CD34 and CD133) and markers characteristic for endothelial cells, such as VEGFR-2 (vascular endothelial growth factor receptor-2), vWF (von Willebrand factor), VE-cadherin (vascular endothelial cadherin) or CD144, Tie-2, CD62E (e-selectin) and c-kit/CD117 [1,7,8,9]. Young, functional EPCs, located mostly in the bone marrow, are particularly characterized by three cell markers: CD133, CD34 and VEGFR-2. After leaving bone marrow and entering peripheral circulation, over time, EPCs lose CD133 and start to express CD31, VE-cadherin and vWF [1]. Based on the character of culture, two types of EPCs are distinguished, namely, early EPCs (e-EPCs) and late EPCs (l-EPCs). Early EPCs, also termed as circulatory angiogenic cells (CACs) or colony-forming unit endothelial cells (CFU-EC), have a low proliferative capacity and secrete various proangiogenic factors [9,10,11,12,13], whereas late EPCs, also called outgrowth endothelial cells (OECs) or endothelial colony-forming cells (ECFCs), possess a high proliferative potential and a capacity to differentiate into mature endothelial cells [9,14,15,16,17,18,19]. Both early and late EPCs have a role in angiogenesis, however, through different mechanisms. Early EPCs encourage angiogenesis in a paracrine way through releasing a variety of pro-angiogenic growth factors [9,10,11,12,13], whereas late EPCs are capable of directly forming new vasculature [20]. Overall, both early and late EPCs are essential in the process of the repair of damaged endothelium [21] (Figure 1).

Endothelial dysfunction is an important component in multiple disorders with neurovascular component, including Alzheimer’s disease (AD), cerebral small vessel disease (CSVD), ischemic stroke (IS) and migraine [22,23,24,25]. Thus, the subject of involvement of EPCs, or their dysfunction, in these diseases has been gaining increasing scientific attention in the recent years. A thorough understanding of functionalities of this type of cells in neurovascular disorders opens the door for the creation of novel therapeutic strategies with EPCs as therapeutic agents. This review aims to thoroughly summarize our current knowledge on the role of EPCs and their potential practical use as a treatment method in conditions with neurovascular involvement, including Alzheimer’s disease, cerebral small vessel disease, ischemic stroke and migraine.

## 2. Alzheimer’s Disease

Studies have demonstrated that previously occurring vascular and endothelial dysfunction lead to the development of Alzheimer’s disease (AD). Comorbid cerebrovascular disease often accompanies AD. It is believed to have an additive effect on cognitive impairment and lower the threshold for dementia [26]. A dysfunction of cerebral vasculature is one of the earliest occurring events in the pathogenesis of AD [27,28]. According to the two-hit hypothesis of AD, first proposed by Zlokovic, vascular pathology appears primary and contributes to Alzheimer’s tau pathology. Vascular risk factors, such as hypertension, diabetes, cardiac disease and/or stroke (hit one) lead to an endothelial dysfunction in the blood–brain barrier (BBB) and a reduction in cerebral blood flow (CBF), which causes oligemia. An endothelial dysfunction of BBB impairs the clearance of amyloid beta (Aβ), whereas oligemia increases production of Aβ, and both processes lead to Aβ accumulation in the brain (hit two). Moreover, endothelial dysfunction within the BBB causes an infiltration of multiple neurotoxic molecules to the brain [29].

Alterations in the number and functionality of EPCs have been observed in patients with AD in a number of studies (Table 1). Results of the research concerning the number of circulating EPCs in AD patients are conflicting. Studies by Maler et al. and Kong et al. demonstrated that AD patients had decreased counts of circulating CD34+ cells and EPCs, respectively, compared to healthy controls [30,31]. On the other hand, research by Lee et al., Breining et al. and Haiyuan et al. found no significant differences in the numbers of circulating EPCs between patients with AD and healthy control groups [32,33,34]. However, a study conducted by Lee et al. found that patients with AD had lower CFU-ECs than risk factor-matched controls [31]. Interestingly, a study by Bigalke et al. showed an increased number of circulating CD34+ cells in patients with early AD, which is the opposite to the results obtained by Maler et al. [30,35]. Moreover, Bigalke et al. found an association between AD and decreased leptin concentration. Moreover, leptin serum levels were a significant predictor for the number of CD34+ cells. The authors concluded that leptin plasma levels and circulating CD34+ progenitor cells could represent an important molecular link between atherosclerotic diseases and AD [35]. However, in a study by Stellos et al., in which patients were divided according to the stages of AD, a significant increase in circulating CD34+/CD133+ and CD34+ progenitor cells was observed among patients with moderate to severe AD compared to healthy elderly controls. Such increase was not observed in patients with mild AD [36]. However, a study conducted by Haiyuan et al., which also differentiated patients between stages of AD, did not find any significant differences in the number of circulating EPCs between patients from mild, moderate and severe AD groups and healthy control group [34]. These discrepancies in results between studies could be explained by differences in the age of subjects since circulating EPCs were found to decrease with age, which is caused by oxidative stress, as a very recent study showed [37]. However, several studies found a correlation between EPCs and cognitive function. In a study by Lee et al., a reduction of CFU-EC was associated with lower cognitive function [32]. Kong et al. found a correlation between a lower number of circulating EPCs and lower cognitive function [31], whereas a study by Stellos et al. indicated that the number of circulating CD34+/CD133+ progenitor cells was significantly inversely correlated with AD patients’ cognitive function. The authors explained their findings as a stage-dependent regulation of circulating CD34+/CD133+ and CD34+ progenitor cells in patients with AD: these cells might be at a lower level in early AD but may be upregulated in moderate and severe AD where they are a part of the tissue healing mechanisms in the AD brain [36].

Several studies have also investigated functional changes in EPCs in AD. A study conducted by Lee et al. showed that CACs isolated from patients with AD demonstrated reduced chemotaxis, reduced paracrine angiogenic activity, increased senescence and changed gene expression patterns compared to CACs from the risk factor-matched control group. The study also found that an addition of high concentration Aβ to the culture of CACs reduced counts of these cells, induced apoptosis and decreased endothelial nitric oxide synthase (eNOS). However, an application of Aβ at a lower concentration to the CACs culture did not reduce their counts. Moreover, CACs isolated from AD patients were found to be more sensible to the cytotoxic effect of Aβ than CACs obtained from risk factor-matched controls. The study concluded that CACs of AD patients possess intrinsic dysfunctions, which adds to our understanding of endothelial vascular pathogenesis of AD [38]. Moreover, a study by Haiyuan et al. demonstrated that circulating EPCs from moderate and severe AD patients showed significantly lower migration and adhesion capability than those isolated from mild AD patients and healthy controls. Based on results, authors suggested an involvement of a decline in reparative function of EPCs in the development of AD [34].

To date, no human studies have been conducted on the use of EPCs in AD; however, therapeutic properties of EPCs in AD have been investigated in a few pre-clinical studies on animal models (Table 2). Safar et al. conducted a study in which they intravenously transplanted bone marrow-derived endothelial progenitor cells (BM-EPCs) to rats with scopolamine-induced AD-like cognitive impairment. This resulted in an improvement of learning and memory deficits, reduction in amyloid plaques, suppression of Aβ, amyloid precursor protein (APP) and p-tau, corrected alterations in neurotransmitter levels, augmented vascular endothelial growth factor (VEGF), nerve growth factor (NGF), brain-derived neurotrophic factor (BDNF) and suppressed proinflammatory tumor necrosis factor-α (TNF-α), interleukin-1β (IL-1β) and an upregulation of interleukin-10 (IL-10) [39]. In turn, Yuan et al. injected EPCs transfected with green fluorescent protein (GFP) adenoviral vectors into APP/PS1 (amyloid precursor protein/presenilin 1) transgenic mice models of AD and wild-type mice. Results revealed an enhanced penetration of exogenous EPCs into the brains of the APP/PS1 transgenic mice than the wild-type mice [40]. In another study conducted by Zhang et al., APP/PS1 transgenic mice were injected with EPCs into the hippocampus. After a hippocampal transplantation of EPCs, mice showed a significant improvement in spatial learning and memory functions, an upregulation of expression of BBB tight junction proteins (ZO-1, Occludin and Claudin-5), an increase in the microvessel density, a decrease in the Aβ senile plaque deposition and a reduction in hippocampal cell apoptosis. Thus, EPCs could seal the BBB, promote angiogenesis, diminish neuronal loss, stimulate the clearance of Aβ and eventually improve cognitive function in AD [41]. These preclinical studies demonstrate that EPCs appear promising as a therapeutical option in AD. In a recent ex vivo study by Heller et al., EPCs transfected with anti-Aβ antibody fragment were demonstrated to secrete this antibody and reduce aggregation of Aβ [42]. Thus, EPCs could also be potentially utilized as a means of drug delivery in AD.

## 3. Cerebral Small Vessel Disease

Endothelial dysfunction has been recognized as the first event that occurs during the pathogenesis of cerebral small vessel disease (CSVD), a primary cause of vascular dementia (VD) [42,43]. Dysfunctional endothelial cells lead to changes in the surrounding cerebral white matter through a secretion of heat shock protein 90α, which hinders oligodendroglial differentiation and, thus, impairs the process of myelination [43]. Moreover, endothelial dysfunction is also related to the impairment of the BBB and a decrease in CBF, and both of these processes are involved in the development of CSVD. An increased permeability of the BBB causes local microhemorrhages and decreases distal blood flow, which leads to an aggravation of the regional ischemia in the brain [23].

Several studies have investigated EPC counts in human individuals with CSVD (Table 3). Results of the research are conflicting in this regard. Early studies demonstrated that CSVD patients had lower levels of circulating EPCs and decreased EPC cluster counts compared to healthy individuals [44,45]. Later studies differentiated patients according to the burden of CSVD and a very recent study divided EPCs into subpopulations according to their surface markers. Overall, elevated levels of EPCs were related to greater CSVD burden [46,47]. However, circulating CD34+ cells were found to be decreased in the above-mentioned group of patients [47]. These findings suggest that EPC levels may serve as potential biomarkers to track the progression of CSVD.

## 4. Ischemic Stroke

Endothelial damage, induced by risk factors, such as hypertension, diabetes and hyperlipidemia, is an important event in the pathophysiology of ischemic stroke (IS) [48]. Endothelial dysfunction plays a key role in the onset of stroke through a promotion of atherosclerosis, thrombosis, a disruption of the BBB, oxidative stress, inflammation and increased vascular tone [49]. Patients undergoing an acute ischemic stroke were found to have a severe endothelial dysfunction during the first 24 h of the event [50]. Moreover, endothelial dysfunction also appears as a consequence of IS. Global ischemia, with or without reperfusion, was found to impair endothelium-dependent vascular tone regulation, whereas focal ischemia impairs endothelium-dependent vasodilatation [51].

EPC levels are decreased, overall, in multiple states that elevate the risk of stroke, such as atherosclerosis or hypertension [52,53]. However, the number of early EPCs (CD133+/VEGFR2+) increases during the acute phase of ischemic stroke, together with angiogenic growth factors VEGF and FGF (fibroblast growth factor). However, EPCs and angiogenic growth factor levels were found to be inversely correlated with inflammatory factors, suggesting an unfavorable impact of inflammation on the survival and differentiation of EPCs [54]. One study indicated that the level of circulating EPCs transiently elevates for some time after an acute stroke; first, it gradually increases up to 1 week after stroke onset, then remains elevated at 2 weeks and returns to baseline at day 28 [55]. An increase in SDF-1α was also noted early after the occurrence of IS [56]. A recent study demonstrated that the EPC level in stroke patients is higher in the 3rd and 12th month post-stroke than within 7 days after stroke. The peak in the EPC count was observed at 12 months after an ischemic event and was significantly higher than in healthy controls. However, EPCs from stroke patients showed impaired functionality measured by tube-formation capability compared to EPCs from healthy individuals [57]. A novel in vitro study demonstrated that the secretome of EPCs derived from stroke patients was found to promote angiogenesis and maturation of new vessels together with restoring the function of the BBB in ischemic conditions [58]. It was also found that the EPC level is inversely correlated with severity of ischemic lesion [59]. Moreover, a higher level of CFU-ECs during the first week after IS predicted better functional outcome and was associated with reduced infarct growth [60], whereas a low level of circulating EPCs measured 48 h after IS predicted severe neurological impairment [61]. Migratory and angiogenic capacities of EPCs were also found to be associated with increased collateral flow during the acute phase of the stroke and increased CBF at day 7 post-stroke. On the other hand, no associations were found between EPCs and hemorrhagic transformation or recanalization [49]. Currently, there is one ongoing clinical trial (NCT02980354) that aims to investigate whether the number and functionalities of circulating EPCs could serve as biomarkers of severity and type (cortical/lacunar) of ischemic stroke [62]. In vitro research has demonstrated that OECs migrate to the place of vascular injury and repair it in order to maintain neurovascular homeostasis at a time of or after an ischemic injury in the brain. OECs were observed to establish an equally tight in vitro model of the BBB as brain microvascular endothelial cells (BMECs), which shows their capacity to form tight junctions. Moreover, OECs were found to have a greater proliferative and migratory capacity than BMECs. An exogenous addition of OECs to an in vitro model of the BBB (established with astrocytes, pericytes and BMECs) repaired the wound scratch-induced on a layer of BMECs in serum-free conditions [63]. Additionally, a very recent study demonstrated that an outgrowth endothelial cell-derived conditioned media (OEC-CM) prevents the damaging effects of TNF-α on the BBB since the levels of TNF-α were found to be significantly elevated on days 2, 7, 30 and 90 after ischemic stroke and TNF-α impairs function and integrity of the BBB, which is the main early cause of death after IS [64].

Several pre-clinical studies, and a recently conducted clinical study, reported favorable outcomes after an administration of EPCs in the treatment of ischemic stroke (Table 4). EPC transplantation after ischemia was demonstrated to encourage angiogenesis and increase capillary density, also in the peri-infarct area, as well as increase cortical blood flow [65,66,67,68,69,70,71]. Moreover, a reduction in the infarct volume was noted following an administration of EPCs [66,67,71]. EPCs were also demonstrated to stimulate neurogenesis and improve neurological outcomes after stroke [65,67,68,71]. One study used embryonic EPCs (eEPCs), and they were found to perform similar to EPCs derived from adult sources [65,66,67,68,69,70,71,72,73,74,75]. Two studies utilized the EPC-derived conditioned media (EPC-CM), which was also reported to have beneficial effects, such as an increase in capillary density or an improvement in the post-ischemia forelimb strength [10,69]. However, no significant improvement in the axonal rewiring was observed in the EPC-CM-treated mice, contrary to those who received EPCs [70]. EPCs transfected with the adiponectin gene, combinations of EPCs with erythropoietin (EPO) or prolonged fasting, were found to be even more beneficial than EPCs alone [72,73,74]. These findings inspired the investigation into the application of EPCs after ischemic stroke in humans. To date, one such study is finished. EPCs were found to be safe; no toxicity events or infusional or allergic reactions were noted after their administration. Patients who received EPCs were found to have fewer adverse events and a higher Scandinavia Stroke Scale (SSS) at a 3-month follow-up compared to the placebo group. However, no differences in mortality and no significant differences in the neurological improvement (except for the higher SSS score in the EPCs group) were noted between the two groups [75]. One currently ongoing clinical trial (NCT02605707) aims to investigate the use of autologous EPCs in the treatment of chronic ischemic stroke. Results of this trial are not yet available [76].

Stem cells are currently considered as a promising therapeutic agent in the treatment of stroke. A guide of recommendations called STEPS (Stem cell Therapeutics as an Emerging Paradigm for Stroke) was developed in order to assure a precise examination of efficacy and safety of this type of cell in preclinical studies. These guidelines aim to prevent the stem cell therapy from being discarded due to failure after reaching clinical studies. Since progenitor cells exhibit similarities to stem cells, the STEPS recommendations could also potentially be applied to the research concerning EPCs. With regards to applying STEPS guidelines to the pre-clinical studies on EPCs in ischemic stroke, certainly more studies on animals of both sexes are needed, as well as studies examining dose–response effects by using different doses of cells within one study in order to determine the optimal dosage. Additionally, applying a well-designed control group is recommended (animals receiving rehabilitation only or treating controls with dead cells and vehicle) [77].

## 5. Migraine

Endothelial dysfunction is also known to be involved in the pathophysiology of migraine. Oxidative stress and inflammation were identified as two main causes of endothelial damage in migraine. Oxidative stress causes a reduction in the amount of nitric oxide (NO), which leads to vasoconstriction. Moreover, NO insufficiency is associated with perception of pain since NO reduces pain by increasing the cyclic guanosine monophosphate (cGMP) level. Moreover, oxidative stress promotes hypercoagulability. As a consequence, endothelial dysfunction leads to increased vascular tone, inflammation and thrombosis, all of which contribute to migraine [24]. Moreover, studies suggest that migraine, particularly migraine with aura, increases the risk of ischemic stroke [78].

Studies have demonstrated that a lower circulating EPC count is observed in migraineurs. A study by Lee et al. indicated that migraine patients (with or without aura) had a reduced number of EPCs compared to healthy controls and patients with tension type headache (TTH). Moreover, patients with migraine with aura showed lower EPC counts than patients with aura-free migraine. Additionally, EPCs isolated from migraineurs showed reduced migration ability and increased cellular senescence compared to EPCs from normal or TTH subjects [79]. A later study by Rodríguez-Osorio et al. confirmed a lower EPC count in migraine patients and, furthermore, indicated that a number of EPCs decreases with time as migraine progresses [80]. Moreover, one study demonstrated that women suffering from migraine with aura exhibited decreased (compared to age-matched healthy women) SDF-1α, which promotes mobilization of the EPCs from the bone marrow. These results suggest that the compensatory up-regulation of SDF-1α as a response to an injury in migraineurs is somehow disrupted, which adds to the evidence for endothelial dysfunction in migraine [81]. Furthermore, among participants of this study, an inverse correlation was found between the level of SDF-1α and CD144+ and activated CD62E+ endothelial microparticles (EMPs), which are markers of endothelial dysfunction [81,82]. Another study showed that female migraineurs with aura have an increased level of EMPs [83]. Furthermore, another study by Oterino et al. observed a higher number of CD62E+EPCs, a marker of endothelial activation, in migraine patients, both with and without aura [84]. A reduction in and a dysfunction of EPCs in migraine patients was suggested as a link between migraine and cardiovascular risk [79,84].

## 6. Conclusions

Alterations in endothelial progenitor cell count and functionalities have been detected in disorders with neurovascular involvement, i.e., Alzheimer’s disease, ischemic stroke and migraine. Overall, a decreased level of EPCs appears to be associated with worse disease outcome, which suggests that they could serve as a prognostic tool. Moreover, EPC functionalities appear to decline with severity of disease. This makes them a potential therapeutic target and therapeutic agent. Furthermore, it opens a possibility of utilizing EPCs as a means of delivery of other therapeutic drugs. So far, existing pre-clinical studies conducted on animal models and the few human clinical trials on the use of EPCs in neurovascular disorders appear promising in terms of efficacy and safety. The next step will be to thoroughly evaluate EPCs in clinical studies including humans.

### Future Directions

Studies investigating circulating EPC counts in different conditions with a neurovascular component should include individuals of different ages, comorbidities and stages of the disease. This would enable to properly assess the changes in EPCs that occur during the course of a given disease independently of additional variables that influence EPC counts. Moreover, levels of different subpopulations of EPCs based on their surface biomarkers should be evaluated. Therapeutic efficacy and safety of EPCs should be thoroughly analyzed at the stage of pre-clinical trials. Most existing studies used the same dose of EPCs in every subject. Utilizing different doses of EPCs within one study would help to investigate the dose–response effects in order to establish the optimal dosage. Moreover, EPCs from embryonic and adult sources should be compared in terms of effectiveness. Animals of different sexes, ages, comorbidities and stages of the disease should be involved in the studies. The further step is to apply EPCs in clinical trials involving humans.

## Figures and Tables

**Table 1:** A summary of studies on number of circulating EPCs in patients with Alzheimer’s disease.

Author (Year)	Mean Age of Patients	Stage of AD	Results
Maler et al. (2006) [30]	Not specified (*n* = 23)	Early AD	**Decreased** counts of circulating CD34+ cells among AD patients (compared to healthy controls). The number of circulating CD34+ was significantly inversely correlated with Aβ 1-42 and Aβ42/40 ratio in cerebrospinal fluid.
Lee et al. (2009) [32]	71.7 ± 7.8 (*n* = 55)	Newly diagnosed AD	**No significant differences** were found in the number of circulating EPCs between patients with AD and risk factor-matched controls. Patients with AD had lower CFU-ECs than risk factor-matched controls. A reduction in CFU-ECs was associated with lower cognitive function.
Stellos et al. (2010) [36]	Patients with mild AD = 73.8 ± 5.4 Patients with moderate to severe AD = 73.2 ± 9.3 (*n* = 45)	17/45 (38%) of patients had mild AD 28/45 (62%) of patients had moderate to severe AD	Significantly **increased** numbers of circulating CD34+ and CD34+/CD133+ progenitor cells were found among individuals with moderate to severe AD compared to healthy controls. No such changes were detected in patients with mild AD. A negative correlation was found between the level of CD34+/CD133+ progenitor cells circulating in the blood and age, cognitive function and SDF-1α plasma level.
Bigalke et al. (2010) [35]	74.3 ± 9.1 (*n* = 41)	Early AD	An **increased** number of circulating CD34+ cells was associated with the presence of AD and showed an inverse correlation with leptin plasma levels.
Kong et al. (2011) [31]	71.4 ± 2.3 (*n* = 30)	Newly diagnosed AD	**Decreased** number of circulating EPCs in AD patients compared to healthy subjects. A correlation between lower number of circulating EPCs and lower cognitive function.
Breining et al. (2016) [33]	83.2 ± 6.4 (*n* = 48)	Not specified	**No significant differences** were found in the number of circulating EPCs between AD patients and control groups.
Haiyuan et al. (2020) [34]	Patients with mild AD = 76.9 ± 12.0 Patients with moderate AD = 77.1 ± 12.3 Patients with severe AD = 81.3 ± 7.3	19/58 (33%) of patients had mild AD 21/58 (36%) of patients had moderate AD 18/58 (31%) of patients had severe AD	**No significant differences** were found in the number of circulating EPCs between four groups (three groups of AD patients according to AD severity and healthy control group).

Abbreviations: Aβ–amyloid beta; AD–Alzheimer’s disease; CFU-ECs–colony-forming unit endothelial cells; EPCs–endothelial progenitor cells; SDF-1α–stromal cell-derived factor-1α.

**Table 2:** A summary of studies on the use of EPCs in AD.

Author (Year)	Subjects	EPCs Dosage	Results
Safar et al. (2016) [39]	Adult male Wistar rats with cognitive impairment induced by daily administration of scopolamine for 6 weeks	2 × 10^6^ of BM-EPCs administered intravenously to the rat tail vein 5 days after the last scopolamine dose	BM-EPCs migrated into the brain of rats, mitigated the accumulation of Aβ and associated histopathological alterations, dulled the increase in hippocampal Aβ and APP, restored the Aβ-degrading neprilysin and downregulated p-tau. They also boosted VEGF, NGF, and BDNF and suppressed the proinflammatory TNF-α and IL-1β. An application of BM-EPCs also resulted in a correction of perturbed neurotransmitter levels, including acetylcholine, dopamine, GABA and glutamate. Improvements in rats’ deficits in learning and memory were also observed.
Yuan et al. (2016) [40]	APP/PS1 transgenic mice	2 × 10^6^ of EPCs transfected with GFP adenoviral vectors administered intravenously into the tail vein	A penetration of exogenous EPCs into the brain was enhanced in the APP/PS1 transgenic mice compared to wild-type mice.
Zhang et al. (2018) [41]	APP/PS1 transgenic mice	4 × 10^5^ of EPCs transplanted into the hippocampus	A transplantation of EPCs enhanced the expression of BBB tight junction proteins, increased the microvessel density, decreased the Aβ plaque deposition and hippocampal cell apoptosis. Moreover, significant improvements were observed in memory functions and spatial learning in mice transplanted with EPCs.

Abbreviations: Aβ–amyloid beta; APP–amyloid precursor protein; APP/PS1–amyloid precursor protein/presenilin 1; BBB–blood–brain barrier; BM-EPCs–bone marrow-derived endothelial progenitor cells; EPCs–endothelial progenitor cells; GABA–gamma-aminobutyric acid; GFP–green fluorescent protein; IL-1β–interleukin-1β; NGF–nerve growth factor; TNF-α–tumor necrosis factor-α; VEGF–vascular endothelial growth factor.

**Table 3:** A summary of studies on number of circulating EPCs in patients with cerebral small vessel disease.

Author (Year)	Mean Age of Patients	Manifestations of CSVD	Results
Rouhl et al. (2009) [44]	64.0 (±11.4) *n* = 42	Lacunar stroke (which occurred at least 2 years prior)	CSVD patients had **lower EPC cluster counts** than healthy controls. EPC cluster formation was inhibited by patient serum.
Rouhl et al. (2012) [45]	65.2 (±9.3) *n* = 32	Lesions in the white matter, microbleeds or asymptomatic lacunar strokes. All patients had hypertension.	CSVD individuals with hypertension exhibited **lower levels of EPCs** than healthy controls.
Kapoor et al. (2021) [46]	69.8 (±7.3) *n*= 64	CSVD burden determined by MRI markers: microbleeds, small lacunes, white matter hyperintesities. Patients were free of stroke and dementia.	**Increased levels of EPCs** and VEGF were related to greater CSVD burden.
Huang et al. (2021) [47]	*n* = 364	Patients with confirmed CSVD	Patients with greater CSVD burden had **decreased level of circulating CD34+ cells and significantly elevated levels of CD34+CD133+ and CD34+CD133+CD309+ cells** compared to those with lower CSVD burden.

Abbreviations: EPCs–endothelial progenitor cells; CSVD–cerebral small vessel disease.

**Table 4:** A summary of finished and ongoing studies on the use of EPCs in ischemic stroke.

Author (Year)/ National Clinical Trial Identifier (Start Year)	Subjects	EPCs Dosage	Results
Shyu et al. (2006) [65]	Adult male rats after 90-min occlusion of middle cerebral artery	∼2 × 10^5^ of peripheral blood hematopoietic stem cells (PBSCs) (CD34+) were stereotaxically injected intracerebrally 7 days after ischemia	Implanted PBSCs differentiated into glial cells, neurons or endothelial vascular cells. Improvement in neurological behavior, increase in neuronal cortical activity, promotion of formation of new vessels, increase in the local cortical blood flow in the ischemic hemisphere.
Ohta et al. (2006) [66]	Adult male rats after 90 min occlusion of the middle cerebral artery	2.5 × 10^5^ of EPCs were administered into internal carotid artery right after ischemia	Administration of EPCs reduced infarct volume and functional neurological deficits.
Di Santo et al. (2009) [10]	Male athymic nude rats subjected to chronic hindlimb ischemia	1 × 10^6^ of EPCs or 250 µL of EPC-CM were administered intramuscularly at 5 sites into the ischemic hindlimb, 3 times within 7 days, 4 weeks after ischemia	Both EPCs and EPC-CM caused an increase in capillary density, enhanced vascular maturation and muscle viability, which was visible in significantly increased hindlimb blood flow and improved muscle performance. Moreover, EPC-CM stimulated the mobilization of the bone marrow-derived EPCs.
Fan et al. (2010) [67]	Adult mice after 1 h of transient middle cerebral artery occlusion	1 × 10^6^ of EPCs injected into jugular vein right after ischemia	A transplantation of EPCs significantly reduced infarct volume 3 days after ischemia and reduced cortex atrophy 4 weeks after ischemia. EPCs also improved neurobehavioral outcomes and increased angiogenesis in the peri-infarction zone.
Moubarik et al. (2011) [68]	Adult male rats after 60 min of the middle cerebral artery transient occlusion	4 × 10^6^ of endothelial colony-forming cells (ECFCs) injected into femoral vein 24 h after ischemia.	A transplantation of ECFCs was associated with a stimulation of neurogenesis, an increase in capillary density and a reduction in apoptotic cell number at the site of an infarct.
Rosell et al. (2013) [69]	Adult male mice subjected to the middle cerebral artery permanent distal occlusion	10^4^ to 2×10^5^ of EPCs or cell-free conditioned media (CM) obtained from EPCs were administered randomly 30–32 h after ischemia.	A significant increase in the density of capillaries and an improvement in the post-ischemia forelimb strength were noted both among mice treated with EPCs and CM. An increase in axonal rewiring was observed among animals treated with EPCs, but not in those treated with CM.
Pellegrini et al. (2013) [73]	Adult male rats subjected to 1 h of transient middle cerebral artery occlusion	5 × 10^6^ of ECFCs intravenously and/or 2500 UI/kg/day for 3 days of EPO intraperitoneally 24 h after ischemia	The combination of ECFSs and EPO was more effective in increasing angiogenesis and neurogenesis and decreasing apoptosis compared to ECFCs or EPO alone. Also the ECFCs+EPO combination was the only treatment that resulted in a complete recovery of neurological function.
Hecht et al. (2014) [70]	Adult male rats subjected to a 3-vessel occlusion (chronic cerebral hypoperfusion)	1 × 10^6^ of embryonic EPCs (eEPCs) intravenously right after occlusion and at day 7 and day 14 after ischemia.	A treatment with eEPCs provided better functional recovery, which was reflected in significant increases in parenchymal capillary density and in vessel diameters in the anterior Circle of Willis, as well as higher number of leptomeningeal anastomoses.
Bai et al. (2015) [71]	Adult male mice subjected to a right middle cerebral artery occlusion induced by a photochemical reaction	1 × 10^6^ of EPCs injected into the ipsilateral internal carotid artery 24 h after ischemia.	In the EPC-treated mice, increased angiogenesis and neurogenesis, activation of eNOS and the expression of BDNF were increased, axonal growth was stimulated. A decrease was noted in infarct volume and neurological deficits.
Xin et al. (2016) [74]	Adult male mice subjected to a permanent left middle cerebral artery occlusion	1 × 10^6^ of EPCs injected into the tail vein right after cerebral ischemia + mice were subjected to prolonged fasting or periodic prolonged fasting after cerebral ischemia	Prolonged fasting significantly enhanced the EPC functions, angiogenesis and mitigated ischemic injury in the brain.
Zhang et al. (2017) [72]	Adult male rats subjected to 2 h of middle cerebral artery occlusion	2 × 10^6^ of EPCs or LV-APN-EPCs (EPCs transfected with the adiponectin gene) were injected intravenously into the tail vein after 2 h of reperfusion	Higher improvements in infarct area, microvessel density, behavioral function and cell apoptosis were observed in the LV-APN-EPCs group than in the EPCs group.
Fang et al. (2019) [75]	18 adult patients with acute cerebral stroke in the middle cerebral artery territory	2.5 × 10^6^ cells/kg body weight of autologous EPCs administered intravenously 4–5 weeks after ischemia and additional 2.5 × 10^6^ cells/kg body weight of EPCs 1 week after initial boosting	No toxicity events or allergic reactions were noted. Patients who received EPCs had fewer serious adverse events compared to the placebo group; however, there was no difference in mortality between the groups. No significant differences were observed in neurological or functional improvement, except for the higher SSS score among the EPCs group at a 3-month follow-up.
NCT02605707 (2015) [76]	12 adult patients with chronic ischemic stroke (which occurred between 6 and 60 months prior)	Autologous EPCs administered intravenously	Not available

Abbreviations: BDNF–brain-derived neurotrophic factor; CM–conditioned media; ECFCs–endothelial colony-forming cells; eEPCs–embryonic endothelial progenitor cells; eNOS–endothelial nitric oxide synthase; EPC-CM–endothelial progenitor cells-derived conditioned media; EPCs–endothelial progenitor cells; EPO–erythropoietin; PBSCs–peripheral blood hematopoietic stem cells.

## Data Availability

Not applicable.

## Abbreviations (In Alphabetical Order)

AD	Alzheimer’s disease
APP	amyloid precursor protein
APP/PS1	amyloid precursor protein/presenilin 1
BBB	blood–brain barrier
BDNF	brain-derived neurotrophic factor
BMECs	brain microvascular endothelial cells
BM-EPCs	bone marrow-derived endothelial progenitor cells
CACs	circulatory angiogenic cells
CBF	cerebral blood flow
CFU-ECs	colony-forming unit endothelial cells
cGMP	cyclic guanosine monophosphate
CM	conditioned media
CSVD	cerebral small vessel disease
ECFCs	endothelial colony-forming cells
EMPs	endothelial microparticles
EPCs	endothelial progenitor cells
EPC-CM	endothelial progenitor cells-derived conditioned media
EPO	erythropoietin
eNOS	endothelial nitric oxide synthase
eEPCs	embryonic endothelial progenitor cells
e-EPCs	early endothelial progenitor cells
FGF	fibroblast growth factor
GFP	green fluorescent protein
IGF-1	insulin-like growth factor 1
IL-1β	interleukin-1β
IL-10	interleukin-10
IS	ischemic stroke
l-EPCs	late EPCs
NGF	nerve growth factor
NO	nitric oxide
OEC-CM	outgrowth endothelial cell-derived conditioned media
OECs	outgrowth endothelial cells
PBSCs	peripheral blood hematopoietic stem cells
SDF-1α	stromal cell-derived factor-1α
SSS	Scandinavia Stroke Scale
TNF-α	tumor necrosis factor-α
TTH	tension type headache
VD	vascular dementia
VE-cadherin	vascular endothelial cadherin
VEGF	vascular endothelial growth factor
VEGFR-2	vascular endothelial growth factor receptor-2
vWF	von Willebrand factor

## References

[1] Hristov M., Erl W., Weber P.C. (2003). Endothelial progenitor cells: Isolation and characterization. Trends Cardiovasc. Med..

[2] Yoder M.C. (2012). Human endothelial progenitor cells. Cold Spring Harb. Perspect. Med..

[3] Ingram D.A., Mead L.E., Moore D.B., Woodard W., Fenoglio A., Yoder M.C. (2005). Vessel wall-derived endothelial cells rapidly proliferate because they contain a complete hierarchy of endothelial progenitor cells. Blood.

[4] Hu C.H., Li Z.M., Du Z.M., Zhang A.X., Rana J.S., Liu D.H., Yang D.Y., Wu G.F. (2010). Expanded human cord blood-derived endothelial progenitor cells salvage infarcted myocardium in rats with acute myocardial infarction. Clin. Exp. Pharm. Physiol..

[5] Imitola J., Raddassi K., Park K.I., Mueller F.J., Nieto M., Teng Y.D., Frenkel D., Li J., Sidman R.L., Walsh C.A. (2004). Directed migration of neural stem cells to sites of CNS injury by the stromal cell-derived factor 1alpha/CXC chemokine receptor 4 pathway. Proc. Natl. Acad. Sci. USA.

[6] He X.Y., Chen Z.Z., Cai Y.Q., Xu G., Shang J.H., Kou S.B., Li M., Zhang H.T., Duan C.Z., Zhang S.Z. (2011). Expression of cytokines in rat brain with focal cerebral ischemia after grafting with bone marrow stromal cells and endothelial progenitor cells. Cytotherapy.

[7] Rouhl R.P., van Oostenbrugge R.J., Damoiseaux J., Tervaert J.W., Lodder J. (2008). Endothelial progenitor cell research in stroke: A potential shift in pathophysiological and therapeutical concepts. Stroke.

[8] Fadini G.P., Losordo D., Dimmeler S. (2012). Critical reevaluation of endothelial progenitor cell phenotypes for therapeutic and diagnostic use. Circ. Res..

[9] Hur J., Yoon C.H., Kim H.S., Choi J.H., Kang H.J., Hwang K.K., Oh B.H., Lee M.M., Park Y.B. (2004). Characterization of two types of endothelial progenitor cells and their different contributions to neovasculogenesis. Arter. Thromb. Vasc. Biol..

[10] Di Santo S., Yang Z., Wyler von Ballmoos M., Voelzmann J., Diehm N., Baumgartner I., Kalka C. (2009). Novel cell-free strategy for therapeutic angiogenesis: In vitro generated conditioned medium can replace progenitor cell transplantation. PLoS ONE.

[11] Ward M.R., Thompson K.A., Isaac K., Vecchiarelli J., Zhang Q., Stewart D.J., Kutryk M.J. (2011). Nitric oxide synthase gene transfer restores activity of circulating angiogenic cells from patients with coronary artery disease. Mol. Ther..

[12] Cheng C.C., Chang S.J., Chueh Y.N., Huang T.S., Huang P.H., Cheng S.M., Tsai T.N., Chen J.W., Wang H.W. (2013). Distinct angiogenesis roles and surface markers of early and late endothelial progenitor cells revealed by functional group analyses. BMC Genom..

[13] Medina R.J., O’Neill C.L., Sweeney M., Guduric-Fuchs J., Gardiner T.A., Simpson D.A., Stitt A.W. (2010). Molecular analysis of endothelial progenitor cell (EPC) subtypes reveals two distinct cell populations with different identities. BMC Med. Genom..

[14] Sieveking D.P., Buckle A., Celermajer D.S., Ng M.K. (2008). Strikingly different angiogenic properties of endothelial progenitor cell subpopulations: Insights from a novel human angiogenesis assay. J. Am. Coll. Cardiol..

[15] Timmermans F., Van Hauwermeiren F., De Smedt M., Raedt R., Plasschaert F., De Buyzere M.L., Gillebert T.C., Plum J., Vandekerckhove B. (2007). Endothelial outgrowth cells are not derived from CD133+ cells or CD45+ hematopoietic precursors. Arterioscler. Thromb. Vasc. Biol..

[16] Glynn J.J., Hinds M.T. (2014). Endothelial outgrowth cells: Function and performance in vascular grafts. Tissue Eng. Part B Rev..

[17] Martin-Ramirez J., Hofman M., van den Biggelaar M., Hebbel R.P., Voorberg J. (2012). Establishment of outgrowth endothelial cells from peripheral blood. Nat. Protoc..

[18] Bou Khzam L., Bouchereau O., Boulahya R., Hachem A., Zaid Y., Abou-Saleh H., Merhi Y. (2015). Early outgrowth cells versus endothelial colony forming cells functions in platelet aggregation. J. Transl. Med..

[19] Reinisch A., Hofmann N.A., Obenauf A.C., Kashofer K., Rohde E., Schallmoser K., Flicker K., Lanzer G., Linkesch W., Speicher M.R. (2009). Humanized large-scale expanded endothelial colony-forming cells function in vitro and in vivo. Blood.

[20] Yoder M.C., Mead L.E., Prater D., Krier T.R., Mroueh K.N., Li F., Krasich R., Temm C.J., Prchal J.T., Ingram D.A. (2007). Redefining endothelial progenitor cells via clonal analysis and hematopoietic stem/progenitor cell principals. Blood.

[21] Hristov M., Erl W., Weber P.C. (2003). Endothelial progenitor cells: Mobilization, differentiation, and homing. Arterioscler. Thromb. Vasc. Biol..

[22] Custodia A., Ouro A., Romaus-Sanjurjo D., Pías-Peleteiro J.M., de Vries H.E., Castillo J., Sobrino T. (2022). Endothelial Progenitor Cells and Vascular Alterations in Alzheimer’s Disease. Front. Aging Neurosci..

[23] Bai T., Yu S., Feng J. (2022). Advances in the Role of Endothelial Cells in Cerebral Small Vessel Disease. Front. Neurol..

[24] Paolucci M., Altamura C., Vernieri F. (2021). The Role of Endothelial Dysfunction in the Pathophysiology and Cerebrovascular Effects of Migraine: A Narrative Review. J. Clin. Neurol..

[25] Tuttolomondo A., Daidone M., Pinto A. (2020). Endothelial Dysfunction and Inflammation in Ischemic Stroke Pathogenesis. Curr. Pharm. Des..

[26] Toledo J.B., Arnold S.E., Raible K., Brettschneider J., Xie S.X., Grossman M., Monsell S.E., Kukull W.A., Trojanowski J.Q. (2013). Contribution of cerebrovascular disease in autopsy confirmed neurodegenerative disease cases in the National Alzheimer’s Coordinating Centre. Brain.

[27] Parodi-Rullán R., Sone J.Y., Fossati S. (2019). Endothelial Mitochondrial Dysfunction in Cerebral Amyloid Angiopathy and Alzheimer’s Disease. J. Alzheimers. Dis..

[28] Corriveau R.A., Bosetti F., Emr M., Gladman J.T., Koenig J.I., Moy C.S., Pahigiannis K., Waddy S.P., Koroshetz W. (2016). The Science of Vascular Contributions to Cognitive Impairment and Dementia (VCID): A Framework for Advancing Research Priorities in the Cerebrovascular Biology of Cognitive Decline. Cell Mol. Neurobiol..

[29] Zlokovic B.V. (2011). Neurovascular pathways to neurodegeneration in Alzheimer’s disease and other disorders. Nat. Rev. Neurosci..

[30] Maler J.M., Spitzer P., Lewczuk P., Kornhuber J., Herrmann M., Wiltfang J. (2006). Decreased circulating CD34+ stem cells in early Alzheimer’s disease: Evidence for a deficient hematopoietic brain support?. Mol. Psychiatry.

[31] Kong X.D., Zhang Y., Liu L., Sun N., Zhang M.Y., Zhang J.N. (2011). Endothelial progenitor cells with Alzheimer’s disease. Chin. Med. J..

[32] Lee S.T., Chu K., Jung K.H., Park H.K., Kim D.H., Bahn J.J., Kim J.H., Oh M.J., Lee S.K., Kim M. (2009). Reduced circulating angiogenic cells in Alzheimer disease. Neurology.

[33] Breining A., Silvestre J.S., Dieudonné B., Vilar J., Faucounau V., Verny M., Néri C., Boulanger C.M., Boddaert J. (2016). Biomarkers of vascular dysfunction and cognitive decline in patients with Alzheimer’s disease: No evidence for association in elderly subjects. Aging Clin. Exp. Res..

[34] Haiyuan L., Xue X., Min L., Lingyu W., Xianlin G., Hancong S., Qiulei C., Jia X. (2020). Study of quantity and function of endothelial progenitor cells in peripheral blood of patients with Alzheimer’s disease. J. New Med..

[35] Bigalke B., Schreitmüller B., Sopova K., Paul A., Stransky E., Gawaz M., Stellos K., Laske C. (2011). Adipocytokines and CD34 progenitor cells in Alzheimer’s disease. PLoS ONE.

[36] Stellos K., Panagiota V., Sachsenmaier S., Trunk T., Straten G., Leyhe T., Seizer P., Geisler T., Gawaz M., Laske C. (2010). Increased circulating progenitor cells in Alzheimer’s disease patients with moderate to severe dementia: Evidence for vascular repair and tissue regeneration?. J. Alzheimers Dis..

[37] Reskiawan A., Kadir R., Alwjwaj M., Ahmad Othman O., Rakkar K., Sprigg N., Bath P.M., Bayraktutan U. (2022). Inhibition of oxidative stress delays senescence and augments functional capacity of endothelial progenitor cells. Brain Res..

[38] Lee S.T., Chu K., Jung K.H., Jeon D., Bahn J.J., Kim J.H., Kun Lee S., Kim M., Roh J.K. (2010). Dysfunctional characteristics of circulating angiogenic cells in Alzheimer’s disease. J. Alzheimers Dis..

[39] Safar M.M., Arab H.H., Rizk S.M., El-Maraghy S.A. (2016). Bone Marrow-Derived Endothelial Progenitor Cells Protect Against Scopolamine-Induced Alzheimer-Like Pathological Aberrations. Mol. Neurobiol..

[40] Yuan X., Mei B., Zhang L., Zhang C., Zheng M., Liang H., Wang W., Zheng J., Ding L., Zheng K. (2016). Enhanced penetration of exogenous EPCs into brains of APP/PS1 transgenic mice. Am. J. Transl. Res..

[41] Zhang S., Zhi Y., Li F., Huang S., Gao H., Han Z., Ge X., Li D., Chen F., Kong X. (2018). Transplantation of in vitro cultured endothelial progenitor cells repairs the blood-brain barrier and improves cognitive function of APP/PS1 transgenic AD mice. J. Neurol. Sci..

[42] Rajani R.M., Quick S., Ruigrok S.R., Graham D., Harris S.E., Verhaaren B.F.J., Fornage M., Seshadri S., Atanur S.S., Dominiczak A.F. (2018). Reversal of endothelial dysfunction reduces white matter vulnerability in cerebral small vessel disease in rats. Sci. Transl. Med..

[43] Pantoni L. (2010). Cerebral small vessel disease: From pathogenesis and clinical characteristics to therapeutic challenges. Lancet Neurol..

[44] Rouhl R.P., van Oostenbrugge R.J., Damoiseaux J.G., Debrus-Palmans L.L., Theunissen R.O., Knottnerus I.L., Staals J.E., Delanghe J.R., Tervaert J.W., Lodder J. (2009). Haptoglobin phenotype may alter endothelial progenitor cell cluster formation in cerebral small vessel disease. Curr. Neurovasc. Res..

[45] Rouhl R.P., Mertens A.E., van Oostenbrugge R.J., Damoiseaux J.G., Debrus-Palmans L.L., Henskens L.H., Kroon A.A., de Leeuw P.W., Lodder J., Tervaert J.W. (2012). Angiogenic T-cells and putative endothelial progenitor cells in hypertension-related cerebral small vessel disease. Stroke.

[46] Kapoor A., Gaubert A., Marshall A., Meier I.B., Yew B., Ho J.K., Blanken A.E., Dutt S., Sible I.J., Li Y. (2021). Increased Levels of Circulating Angiogenic Cells and Signaling Proteins in Older Adults With Cerebral Small Vessel Disease. Front. Aging Neurosci..

[47] Huang Z.X., Fang J., Zhou C.H., Zeng J., Yang D., Liu Z. (2021). CD34+ cells and endothelial progenitor cell subpopulations are associated with cerebral small vessel disease burden. Biomark Med..

[48] Heller L., Thinard R., Chevalier M., Arpag S., Jing Y., Greferath R., Heller R., Nicolau C. (2020). Secretion of proteins and antibody fragments from transiently transfected endothelial progenitor cells. J. Cell Mol. Med..

[49] Sargento-Freitas J., Aday S., Nunes C., Cordeiro M., Gouveia A., Silva F., Machado C., Rodrigues B., Santo G.C., Ferreira C. (2018). Endothelial Progenitor Cells influence acute and subacute stroke hemodynamics. J. Neurol. Sci..

[50] Hu X., De Silva T.M., Chen J., Faraci F.M. (2017). Cerebral Vascular Disease and Neurovascular Injury in Ischemic Stroke. Circ. Res..

[51] Blum A., Vaispapir V., Keinan-Boker L., Soboh S., Yehuda H., Tamir S. (2012). Endothelial dysfunction and procoagulant activity in acute ischemic stroke. J. Vasc. Interv. Neurol..

[52] Schmidt-Lucke C., Rössig L., Fichtlscherer S., Vasa M., Britten M., Kämper U., Dimmeler S., Zeiher A.M. (2005). Reduced number of circulating endothelial progenitor cells predicts future cardiovascular events: Proof of concept for the clinical importance of endogenous vascular repair. Circulation.

[53] Umemura T., Soga J., Hidaka T., Takemoto H., Nakamura S., Jitsuiki D., Nishioka K., Goto C., Teragawa H., Yoshizumi M. (2008). Aging and hypertension are independent risk factors for reduced number of circulating endothelial progenitor cells. Am. J. Hypertens..

[54] Golab-Janowska M., Paczkowska E., Machalinski B., Kotlega D., Meller A., Safranow K., Wankowicz P., Nowacki P. (2019). Elevated Inflammatory Parameter Levels Negatively Impact Populations of Circulating Stem Cells (CD133+), Early Endothelial Progenitor Cells (CD133+/VEGFR2+), and Fibroblast Growth Factor in Stroke Patients. Curr. Neurovasc. Res..

[55] Zhou W.J., Zhu D.L., Yang G.Y., Zhang Y., Wang H.Y., Ji K.D., Lu Y.M., Gao P.J. (2009). Circulating endothelial progenitor cells in Chinese patients with acute stroke. Hypertens. Res..

[56] Bogoslovsky T., Spatz M., Chaudhry A., Maric D., Luby M., Frank J., Warach S. (2011). NINDS Natural History of Stroke Investigators. Stromal-derived factor-1[alpha] correlates with circulating endothelial progenitor cells and with acute lesion volume in stroke patients. Stroke.

[57] Kukumberg M., Zaw A.M., Wong D.H.C., Toh C.M., Chan B.P.L., Seet R.C.S., Wong P.T.H., Yim E.K.F. (2021). Characterization and Functional Assessment of Endothelial Progenitor Cells in Ischemic Stroke Patients. Stem Cell Rev. Rep..

[58] Loiola R.A., García-Gabilondo M., Grayston A., Bugno P., Kowalska A., Duban-Deweer S., Rizzi E., Hachani J., Sano Y., Shimizu F. (2021). Secretome of endothelial progenitor cells from stroke patients promotes endothelial barrier tightness and protects against hypoxia-induced vascular leakage. Stem Cell Res. Ther..

[59] Bogoslovsky T., Chaudhry A., Latour L., Maric D., Luby M., Spatz M., Frank J., Warach S. (2010). Endothelial progenitor cells correlate with lesion volume and growth in acute stroke. Neurology.

[60] Sobrino T., Hurtado O., Moro M.A., Rodríguez-Yáñez M., Castellanos M., Brea D., Moldes O., Blanco M., Arenillas J.F., Leira R. (2007). The increase of circulating endothelial progenitor cells after acute ischemic stroke is associated with good outcome. Stroke.

[61] Yip H.K., Chang L.T., Chang W.N., Lu C.H., Liou C.W., Lan M.Y., Liu J.S., Youssef A.A., Chang H.W. (2008). Level and value of circulating endothelial progenitor cells in patients after acute ischemic stroke. Stroke.

[62] Rakkar K., Othman O., Sprigg N., Bath P., Bayraktutan U. (2020). Endothelial progenitor cells, potential biomarkers for diagnosis and prognosis of ischemic stroke: Protocol for an observational case-control study. Neural. Regen. Res..

[63] Abdulkadir R.R., Alwjwaj M., Othman O.A., Rakkar K., Bayraktutan U. (2020). Outgrowth endothelial cells form a functional cerebral barrier and restore its integrity after damage. Neural. Regen. Res..

[64] Kadir R.R.A., Alwjwaj M., Rakkar K., Othman O.A., Sprigg N., Bath P.M., Bayraktutan U. (2022). Outgrowth Endothelial Cell Conditioned Medium Negates TNF-α-Evoked Cerebral Barrier Damage: A Reverse Translational Research to Explore Mechanisms. Stem Cell Rev. Rep..

[65] Shyu W.C., Lin S.Z., Chiang M.F., Su C.Y., Li H. (2006). Intracerebral peripheral blood stem cell (CD34+) implantation induces neuroplasticity by enhancing beta1 integrin-mediated angiogenesis in chronic stroke rats. J. Neurosci..

[66] Ohta T., Kikuta K., Imamura H., Takagi Y., Nishimura M., Arakawa Y., Hashimoto N., Nozaki K. (2006). Administration of ex vivo-expanded bone marrow-derived endothelial progenitor cells attenuates focal cerebral ischemia-reperfusion injury in rats. Neurosurgery.

[67] Fan Y., Shen F., Frenzel T., Zhu W., Ye J., Liu J., Chen Y., Su H., Young W.L., Yang G.Y. (2010). Endothelial progenitor cell transplantation improves long-term stroke outcome in mice. Ann. Neurol..

[68] Moubarik C., Guillet B., Youssef B., Codaccioni J.L., Piercecchi M.D., Sabatier F., Lionel P., Dou L., Foucault-Bertaud A., Velly L. (2011). Transplanted late outgrowth endothelial progenitor cells as cell therapy product for stroke. Stem Cell Rev. Rep..

[69] Rosell A., Morancho A., Navarro-Sobrino M., Martínez-Saez E., Hernández-Guillamon M., Lope-Piedrafita S., Barceló V., Borrás F., Penalba A., García-Bonilla L. (2013). Factors secreted by endothelial progenitor cells enhance neurorepair responses after cerebral ischemia in mice. PLoS ONE.

[70] Hecht N., Schneider U.C., Czabanka M., Vinci M., Hatzopoulos A.K., Vajkoczy P., Woitzik J. (2014). Endothelial progenitor cells augment collateralization and hemodynamic rescue in a model of chronic cerebral ischemia. J. Cereb. Blood Flow Metab..

[71] Bai Y.Y., Peng X.G., Wang L.S., Li Z.H., Wang Y.C., Lu C.Q., Ding J., Li P.C., Zhao Z., Ju S.H. (2015). Bone Marrow Endothelial Progenitor Cell Transplantation After Ischemic Stroke: An Investigation Into Its Possible Mechanism. CNS Neurosci. Ther..

[72] Zhang R., Xie X., Yu Q., Feng H., Wang M., Li Y., Liu Y. (2017). Constitutive Expression of Adiponectin in Endothelial Progenitor Cells Protects a Rat Model of Cerebral Ischemia. Neural. Plast..

[73] Pellegrini L., Bennis Y., Guillet B., Velly L., Garrigue P., Sabatier F., Dignat-George F., Bruder N., Pisano P. (2013). Therapeutic benefit of a combined strategy using erythropoietin and endothelial progenitor cells after transient focal cerebral ischemia in rats. Neurol. Res..

[74] Xin B., Liu C.L., Yang H., Peng C., Dong X.H., Zhang C., Chen A.F., Xie H.H. (2016). Prolonged Fasting Improves Endothelial Progenitor Cell-Mediated Ischemic Angiogenesis in Mice. Cell Physiol. Biochem..

[75] Fang J., Guo Y., Tan S., Li Z., Xie H., Chen P., Wang K., He Z., He P., Ke Y. (2019). Autologous Endothelial Progenitor Cells Transplantation for Acute Ischemic Stroke: A 4-Year Follow-Up Study. Stem Cells Transl. Med..

[76] Autologous Endothelial Progenitor Cells Transplantation for Chronic Ischemic Stroke—Full Text View—Clinicaltrials.Gov. https://www.clinicaltrials.gov/ct2/show/NCT02605707.

[77] Diamandis T., Borlongan C.V. (2015). One, two, three steps toward cell therapy for stroke. Stroke.

[78] Hu X., Zhou Y., Zhao H., Peng C. (2017). Migraine and the risk of stroke: An updated meta-analysis of prospective cohort studies. Neurol. Sci..

[79] Lee S.T., Chu K., Jung K.H., Kim D.H., Kim E.H., Choe V.N., Kim J.H., Im W.S., Kang L., Park J.E. (2008). Decreased number and function of endothelial progenitor cells in patients with migraine. Neurology.

[80] Rodríguez-Osorio X., Sobrino T., Brea D., Martínez F., Castillo J., Leira R. (2012). Endothelial progenitor cells: A new key for endothelial dysfunction in migraine. Neurology.

[81] Liman T.G., Neeb L., Rosinski J., Reuter U., Endres M. (2016). Stromal Cell-Derived Factor-1 Alpha Is Decreased in Women with Migraine with Aura. Headache.

[82] Leite A.R., Borges-Canha M., Cardoso R., Neves J.S., Castro-Ferreira R., Leite-Moreira A. (2020). Novel Biomarkers for Evaluation of Endothelial Dysfunction. Angiology.

[83] Liman T.G., Bachelier-Walenta K., Neeb L., Rosinski J., Reuter U., Böhm M., Endres M. (2015). Circulating endothelial microparticles in female migraineurs with aura. Cephalalgia.

[84] Oterino A., Toriello M., Palacio E., Quintanilla V.G., Ruiz-Lavilla N., Montes S., Vega M.S., Martinez-Nieto R., Castillo J., Pascual J. (2013). Analysis of endothelial precursor cells in chronic migraine: A case-control study. Cephalalgia.

